# Populations in environments with a soft carrying capacity are eventually extinct

**DOI:** 10.1007/s00285-020-01527-5

**Published:** 2020-08-20

**Authors:** Peter Jagers, Sergei Zuyev

**Affiliations:** Department of Mathematical Sciences, Chalmers University of Technology and University of Gothenburg, SE-412 96, Gothenburg, Sweden

**Keywords:** Population dynamics, Extinction, Martingales, Stochastic stability, 92D25, 60G42, 60K40

## Abstract

Consider a population whose size changes stepwise by its members reproducing or dying (disappearing), but is otherwise quite general. Denote the initial (non-random) size by $Z_0$ and the size of the *n*th change by $C_n$, $n= 1, 2, \ldots$. Population sizes hence develop successively as $Z_1=Z_0+C_1,\ Z_2=Z_1+C_2$ and so on, indefinitely or until there are no further size changes, due to extinction. Extinction is thus assumed final, so that $Z_n=0$ implies that $Z_{n+1}=0$, without there being any other finite absorbing class of population sizes. We make no assumptions about the time durations between the successive changes. In the real world, or more specific models, those may be of varying length, depending upon individual life span distributions and their interdependencies, the age-distribution at hand and intervening circumstances. We could consider toy models of Galton–Watson type generation counting or of the birth-and-death type, with one individual acting per change, until extinction, or the most general multitype CMJ branching processes with, say, population size dependence of reproduction. Changes may have quite varying distributions. The basic assumption is that there is a *carrying capacity*, i.e. a non-negative number *K* such that the conditional expectation of the change, given the complete past history, is non-positive whenever the population exceeds the carrying capacity. Further, to avoid unnecessary technicalities, we assume that the change $C_n$ equals -1 (one individual dying) with a conditional (given the past) probability uniformly bounded away from 0. It is a simple and not very restrictive way to avoid parity phenomena, it is related to irreducibility in Markov settings. The straightforward, but in contents and implications far-reaching, consequence is that all such populations must die out. Mathematically, it follows by a supermartingale convergence property and positive probability of reaching the absorbing extinction state.

## “All surnames tend to be lost”

Almost a century and a half have passed since Galton (1873) and Galton and Watson (1875) introduced their famous simple branching process followed by the infamous conclusion that all families (“surnames”) must die out: “All surnames tend to extinction [...] and this result might have been anticipated, for a surname lost can never be recovered.” Since long it is textbook knowledge, that the extinction probability of supercritical Galton–Watson (and more general) branching processes is less than one, the alternative to extinction being unbounded exponential growth. For a loose discussion of this dichotomy and reflections on what circumstances that might salvage Galton’s and Watson’s conclusion, see, e.g., Haccou et al. (2005). Here we prove almost sure extinction of quite general, stepwise changing populations, which can reach any size but live in a habitat with a carrying capacity, interpreted as a border line where reproduction becomes sub-critical, but which may be crossed by population size, i.e. a *soft*, carrying capacity.

Mathematically, what happens is that the population size process becomes a super-martingale, when crossing the carrying capacity, and extinction follows from a combination of martingale properties. In the population dynamics context, the result is fundamental and applies broadly, e.g., to Markov and to general population size-dependent branching processes as discussed by Jagers and Klebaner (2011), provided the conditional survival times and reproduction processes, given the past satisfy continuity and conditional independence conditions.

The concept of a soft carrying capacity, strictly defined in Assumption 1 beneath, is new but not unrelated to earlier discussion in biological and mathematical population dynamics on ideas of density dependence, see, e.g., papers by Ginzburg et al. (1990), Berryman (1991) and Nisbet and Bence (1989).

## Dynamics of population changes

Consider a population which starts from a non-random number $Z_0$ of individuals. These can be of various types and ages, we shall not go into details. Changes occur successively by the death or reproduction of the population members, and are denoted $C_n,\ n\in \mathbb {N}$, where $\mathbb {N}$ stands for the set of positive integers, and *C* for change. Each size change is thus an integer valued random variable. After the first change, there are $Z_1=Z_0+C_1$ individuals present, and generally $Z_{n+1} = Z_n +C_{n+1}$, as long as $Z_n>0$. If $Z_n=0$, then so is $Z_{n+1}$. The population has died out. We do not make any assumptions about the time between changes, which in real life or more detailed models may be quite varying and influenced by many factors, external or internal, like the population size or the age-distribution of individuals in the population. Nor are there any assumptions of customary kind about the distributions of or interdependencies between the various $C_n$’s. Without loss of generality, we may assume that $\mathbb {P}(C_n=0\Big |\mathcal {F}_{n-1})=0$: one can always change the indices *n* to correspond solely to non-zero changes in the population size, of course with the corresponding change in the conditional distributions of the size and the time to the next event.

Simultaneous deaths of a few individuals are not excluded, but this must not always be the case: we later assume that with positive probability only *one* individual in the population is dying at a given index *n*. This will always be satisfied by systems where, somewhat vaguely, individual lifespans have jointly continuous distributions and bearings occur in a point process with a finite intensity.

As a somewhat more precise example, satisfying our requirements, consider a general (CMJ) branching process inspired setup, where individuals have independent identically distributed life spans with a continuous distribution function. Assume that during life individuals give birth according to a point process whose intensity may be both population-size-dependent and influenced by maternal age. A classical type of simple processes meeting such requirements are those of birth-and-death processes.

Another interesting case is that of a cell population, where cells evolve in cycles, completed cycles are ended by mitotic division. The cycles may be dependent, but with a positive probability only one cell divides: the size change is either $-1$ (if the cell dies before completing its cycle) or +1 (if two fresh cells replace the mother after mitosis). A “division” resulting in just one daughter cell, sometimes referred to as “asymmetrical”, would thus have to be interpreted either as a division closely followed by the death of one of the daughters, or just the mother cell living on, i.e. no change in numbers (which however would have repercussions on the assumptions for life span distributions).

A toy model, inspired by the Galton–Watson or Moran process, would be to let the changes $C_{n+1}$ occur at the real time points $n=1, 2,\ldots$ by a (somehow chosen) individual either dying or being replaced by two or more individuals, according to a distribution that might depend upon the population size $Z_n$.

## Carrying capacities and extinction

We denote the sigma-algebra of all events up to and including the *n*th occurrence by $\mathcal {F}_n$, and introduce a *carrying capacity* $K>0$, thought of as a large natural number. Being a carrying capacity of the population means that the conditional expectation of the impending change, given its past, satisfies

### Assumption 1

1 $$\begin{aligned} {{\,\mathrm{{\mathbb E}}\,}}[C_{n+1}|\mathcal {F}_n] \le 0, \quad \mathrm {if}\quad Z_n \ge K. \end{aligned}$$

Thus, the carrying capacity, as mentioned, does not provide a categorical barrier: population size may exceed it but then it tends to decrease. For individual based models with a carrying capacity, see, e.g., Fan et al. (2020) or Jagers and Klebaner (2011).

The super-martingale property (1) of the population size process is one basic leg of our analysis, the other being the fact that each individual, whatever the circumstances, always runs a definite risk of death unrelated to the others. Specifically, denoting by $\mathbb {Z}_+$ the set of non-negative natural numbers, we make

### Assumption 2

There is an $\epsilon >0$ such that

2 $$\begin{aligned} \mathbb {P}(C_{n+1}=-1|\mathcal {F}_n) \ge \epsilon \quad \mathrm {for all}\quad n\in \mathbb {Z}_+\,. \end{aligned}$$

This is a technical assumption which in many models can be relaxed. Its purpose is to avoid traps when the system gets into a subset of states not containing zero without possibility to leave it. Or the parity phenomenon when, for instance, the initial number $Z_0$ of individuals is odd but only the changes $C_n$ by even numbers have non-zero probabilities: obviously, such a population will never get extinct. In a Markovian setting, the assumption guarantees that the chain is irreducible.

Define $\nu _1$ to be the first visit of the process below the carrying capacity,

$$\begin{aligned} \nu _1 :=\inf \{n\in \mathbb {Z}_+;\ Z_n<K\}, \end{aligned}$$

Hence, if $0<Z_0<K$, $\nu _1=0$ and $Z_{\nu _1} =Z_0$ , whereas

$$\begin{aligned} Z_0 \ge K\Rightarrow 1\le \nu _1\le \infty \text{ and } Z_{\nu _1} \le K-1, \end{aligned}$$

provided $$\nu _1<\infty $$.

### Lemma 1

$\{Z_{n\wedge \nu _1}\}$ is a non-negative supermartingale whose expectation is bounded by $Z_0$.

### Proof

Since $\nu _1$ is a stopping time, it holds for any $n\in \mathbb {Z}_+$ that

$$\begin{aligned} {{\,\mathrm{{\mathbb E}}\,}}[Z_{(n+1)\wedge \nu _1}|\mathcal {F}_n]= & {} {{\,\mathrm{{\mathbb E}}\,}}[Z_{(n+1)\wedge \nu _1}{{\,\mathrm{{ 1I}}\,}}_{\nu _1 \le n} |\mathcal {F}_n]+ {{\,\mathrm{{\mathbb E}}\,}}[Z_{(n+1)\wedge \nu _1}{{\,\mathrm{{ 1I}}\,}}_{\nu _1> n} |\mathcal {F}_n] \\= & {} {{\,\mathrm{{\mathbb E}}\,}}[Z_{\nu _1}{{\,\mathrm{{ 1I}}\,}}_{\nu _1 \le n}|\mathcal {F}_n] + {{\,\mathrm{{\mathbb E}}\,}}[Z_{n+1}{{\,\mathrm{{ 1I}}\,}}_{\nu _1> n}|\mathcal {F}_n] \\\le & {} Z_{\nu _1}{{\,\mathrm{{ 1I}}\,}}_{\nu _1 \le n} +Z_n{{\,\mathrm{{ 1I}}\,}}_{\nu _1 > n} =Z_{n\wedge \nu _1}. \end{aligned}$$

Hence, the process $(Z_{n\wedge \nu _1})$ converges almost surely (and in $L^1$). Since further $|Z_{n+1}-Z_n|\ge 1$, then on the event $\{\nu _1=\infty \}$ the sequence $(Z_{n\wedge \nu _1}) = (Z_n)$ diverges. Thus

$$\begin{aligned} \mathbb {P}(\nu _1=\infty ) \le \mathbb {P}((Z_{n\wedge \nu _1}) \text { does not converge})=0, \end{aligned}$$

and $$Z_{n\wedge \nu _1}\rightarrow Z_{\nu _1}\le Z_0 \wedge (K-1)$$ a.s.

Continue to define $\mu _1:= \inf \{n>\nu _1;\ Z_n\ge K\}\le \infty$, and proceed recursively to

$$\begin{aligned} \nu _{k+1}&:= \inf \{n>\mu _k;\ Z_n<K\},\quad k\in \mathbb {N}, \\ \mu _k&:= \inf \{n>\nu _k;\ Z_n\ge K\},\quad k \in \mathbb {N}, \end{aligned}$$

indefinitely or until one of the $\nu _k$ is infinity. $\mathbb {N}$, as usual, stands for the set of natural numbers. Clearly, extinction $\{Z_n=0\}$ must occur after the last $\nu _k<\infty$, if there is any.

### Theorem 1

Under the two basic assumptions (1) and (2) made, of a carrying capacity and a definite individual death risk, $\mathbb {P}(Z_n \rightarrow 0)=1$, i.e. extinction is (almost) certain.

### Proof

If $\nu _k<\infty$ then so is $\mu _k$, unless the population dies out before reaching or passing *K*. Denote $Z_{\nu _k}:=z_k$ for short. Then

$$\begin{aligned} \mathbb {P}(\mu _k=\infty \Big |\mathcal {F}_{\nu _k})\ge \mathbb {P}(C_{\nu _k+1}=-1,\quad C_{\nu _k+2}=-1,\dotsc ,\quad C_{\nu _k+z_k}=-1\Big |\mathcal {F}_{\nu _k}). \end{aligned}$$

Using (2) and the tower property of conditional expectations,

$$\begin{aligned} \mathbb {P}(C_{\nu _k+1}=-1, C_{\nu _k+2}= & {} -1\Big |\mathcal {F}_{\nu _k})= {{\,\mathrm{{\mathbb E}}\,}}\big [{{\,\mathrm{{\mathbb E}}\,}}[{{\,\mathrm{{ 1I}}\,}}_{C_{\nu _k+1}=-1} {{\,\mathrm{{ 1I}}\,}}_{C_{\nu _k+2}=-1}\Big |\mathcal {F}_{\nu _k+1}]\Big |\mathcal {F}_{\nu _k}\big ]\\= & {} {{\,\mathrm{{\mathbb E}}\,}}\big [{{\,\mathrm{{ 1I}}\,}}_{C_{\nu _k+1}=-1} {{\,\mathrm{{\mathbb E}}\,}}[{{\,\mathrm{{ 1I}}\,}}_{C_{\nu _k+2}=-1}\Big |\mathcal {F}_{\nu _k+1}]\Big |\mathcal {F}_{\nu _k}\big ]\\\ge & {} \epsilon {{\,\mathrm{{\mathbb E}}\,}}[{{\,\mathrm{{ 1I}}\,}}_{C_{\nu _k+1}=-1}\Big |\mathcal {F}_{\nu _k}]\ge \epsilon ^2, \end{aligned}$$

and so on, leading to

$$\begin{aligned} \mathbb {P}(\mu _{k}<\infty |\nu _{k}<\infty )\le 1-\epsilon ^{z_k} \ge p:=1-\epsilon ^{K-1} \end{aligned}$$

because $z_k=Z_{\nu _k}\le K-1$. By the supermartingale property (1), $(Z_n)$ must return (almost) always below *K* from a level equal to or above the carrying capacity. Hence, almost surely

$$\begin{aligned} \nu _{k+1}<\infty \Leftrightarrow \mu _{k}<\infty ,\quad k=1,2,\ldots \end{aligned}$$

Since the sequence $(\mu _k)$ does not decrease, it follows that

$$\begin{aligned} \mathbb {P}(\mu _{k}<\infty )= & {} \mathbb {P}(\mu _{k}<\infty \Big |\mu _{k-1}<\infty )\,\mathbb {P}(\mu _{k-1}<\infty )=\\= & {} \mathbb {P}(\mu _{k}<\infty \Big |\nu _{k}<\infty )\,\mathbb {P}(\mu _{k-1}<\infty )\\\le & {} p\, \mathbb {P}(\mu _{k-1}<\infty )\le \ldots \le p^k\rightarrow 0. \end{aligned}$$

Hence,

$$\begin{aligned} \mathbb {P}(\exists k:\ \mu _{k}=\infty )= \lim _{k\rightarrow \infty }\mathbb {P}(\mu _{k}=\infty )=1. \end{aligned}$$

Qualitatively, depending on the starting state, the population either gets extinct quickly or evolves below and around the carrying capacity *K* until it eventually dies out. The population size, although unbounded, does not get much larger than *K*. Indeed, from a supermartingale form of Doob’s maximal inequality, see, e.g., Corollary 2.4.6 in the book by Menshikov et al. (2016),

$$\begin{aligned} \mathbb {P}\big (\max _{n\ge 0} Z_{(\mu _{k-1}+n)\wedge \nu _k}\ge x\Big |\mathcal {F}_{\mu _{k-1}}\big )\le \frac{K-1}{x} \end{aligned}$$

of course non-trivial only for $x\ge K$.

Although extinction is almost certain, the number of steps to it may, however, be quite large. For instance, when *K* is big and $Z_n$ is a submartingale (i.e. ${{\,\mathrm{{\mathbb E}}\,}}[C_{n+1}|\mathcal {F}_n] \ge 0$) on the set $\{Z_n<K\}$, the system of size $K-1$ needs to go a long way against a non-negative drift to reach 0. Applying Doob’s maximal inequality to the supermartingale $X_n=K-Z_{(\nu _{k}+n)\wedge \mu _k}$ with $X_0=1$, we have that

$$\begin{aligned} \mathbb {P}(Z_{\nu _k+n\wedge \mu _k}=0\Big |\mathcal {F}_{\nu _k})=\mathbb {P}\big (\max _{n\ge 0} X_n\ge K\Big |\mathcal {F}_{\nu _k}\big )\le \frac{1}{K} \end{aligned}$$

so it takes on average at least *K* excursions to the domain below the capacity to die out. In the general case we consider, nothing more can be said: our model includes, as a particular example, the symmetric simple random walk for which the maximal inequality is sharp. But under additional assumptions, the average number of excursions and time to extinction may grow exponentially in *K* (cf. the exponential lower bound on the extinction in the Proof of Theorem 1 above). For instance, this is the case when the increments $C_n$ are totally bounded and the mean drift below *K* is strictly positive: ${{\,\mathrm{{\mathbb E}}\,}}[C_{n+1}\Big |\mathcal {F}_n]\ge \delta$ almost surely for some $\delta >0$ and $0<Z_n<K$ , see, e.g., Theorem 2.5.14 by Menshikov et al. (2016). Similarly, in the presence of a strictly negative drift above the carrying capacity, ${{\,\mathrm{{\mathbb E}}\,}}[C_{n+1}\Big |\mathcal {F}_n]\le -\delta$ almost surely for $Z_n> K$, by Theorem 2.6.2 in the same book, we have that ${{\,\mathrm{{\mathbb E}}\,}}[\nu _k]\le K/\delta$ for all $k\in \mathbb {N}$. If, in addition, $C_n$ are totally bounded then according to Theorem 2.5.14 there, the probability for the population to reach size $K+x$ during an excursion above the capacity decays at least exponentially in *x*. Qualitatively, in the presence of the drifts uniformly separated from 0, the population size bounces around the carrying capacity *K* for quite a long (the time scales exponentially with *K*) before eventually dying out.

Note also, that the uniform positivity condition in (2) is necessary for the imminent extinction: it is easy to produce examples when $\mathbb {P}(C_{n+1}=-1\Big |Z_n=1)$ decays with *n* so quickly that with positive probability the jump to 0 never happens although the absorbing state 0 remains attainable with positive probability.
